# HPLC-UV/Vis-APCI-MS/MS Determination of Major Carotenoids and Their Bioaccessibility from “Delica” (*Cucurbita maxima*) and “Violina” (*Cucurbita moschata*) Pumpkins as Food Traceability Markers

**DOI:** 10.3390/molecules23112791

**Published:** 2018-10-27

**Authors:** Caterina Bergantin, Annalisa Maietti, Paola Tedeschi, Guillermina Font, Lara Manyes, Nicola Marchetti

**Affiliations:** 1 Department of Chemistry and Pharmaceutical Sciences, University of Ferrara, via L. Borsari 46, 44121 Ferrara, Italy; caterina.bergantin@unife.it (C.B.); annalisa.maietti@unife.it (A.M.); paola.tedeschi@unife.it (P.T.); 2 Laboratory of Food Chemistry and Toxicology, Faculty of Pharmacy, University of Valencia, Av. Vicent Andres Estelles s/n, 46100 Burjassot, Spain; guillermina.font@uv.es (G.F.); lara.manyes@uv.es (L.M.)

**Keywords:** carotenoids, pumpkins, HPLC-UV/Vis-APCI-MS/MS, C30-column, bioaccessibility

## Abstract

Carotenoids are a widespread group of fat-soluble pigments, and their major nutritional importance comes from their pro-vitamin A activity and their antioxidant capacity. In this study, two different pumpkin cultivars (*Cucurbita maxima*, also named ‘Delica’ and *Cucurbita moschata*, also known as ‘Violina’) from the southern Po Delta area were investigated in terms of carotenoid content and the influence of food processing on compositional changes and carotenoid bioaccessibility. Quali- and quantitative determination of carotenoids in sample extracts were performed on a C30 column by means of an online coupled HPLC-UV/Vis-APCI-MS/MS technique. The identification of separated compounds was tentatively achieved by merging (i) chromatographic data, (ii) UV-Vis spectra, and (iii) MS/MS fragmentation spectra. The chromatographic profiles for the two cultivars showed qualitative differences. Two major carotenoids were considered for quantification purposes and further investigations: lutein and β-carotene. Quantification of target carotenoids was performed with external calibration through analytical standards. The concentration of lutein and β-carotene was higher in *C. maxima* than in the other variety, *C. moschata*. Carotenoids are susceptible to degradation (isomerization and oxidation) during food processing (i.e., cooking), and the concentration of lutein and β-carotene were monitored in oven-cooked and steam-cooked pumpkins. The steam-cooking process was superior in terms of limiting carotenoid loss. A complete functional profile of pumpkins as a source of carotenoids was gained with the evaluation of their in vitro bioaccessibility and their bioavailability after intake during human digestion. Bioaccessibility of lutein and β-carotene were estimated by an in vitro static digestion model that involved salivary, gastric, and duodenal phases. Bioaccessibility values progressively increased from the salivary to the duodenal phase for both pumpkin varieties and cooking methods. Bioaccessibility of lutein was always lower than β-carotene for both cultivars and for both cooking methods. Bioaccessibility values for lutein and β-carotene changed from 1.93% to 2.34% vs. 4.94% and 8.83% in the salivary phase, from 2.7% to 4.63% vs. 7.83% and 15.60% in the gastric phase, and from 10.04% to 13.42% vs. 25.81% and 35.32% in the duodenal phase. For both target compounds, bioaccessibility in the duodenal phase was more than twice the gastric values, and it underlined that the type of cooking did not influence release from the initial matrix.

## 1. Introduction

Carotenoids are fat-soluble plant pigments associated with lipid fractions due to their hydrophobicity. These compounds are generally made up of two C${}_{20}$ geranylgeranyl diphosphate units joined by tail-to-tail bonds. Most carotenoids have one or two ring structures formed by the cyclization of the end groups. In addition to carbon and hydrogen, they may also contain oxygen atoms. The most characteristic property of the carotenoid structure is the long system of alternating double and single bonds. This forms a conjugated system in the central part of the molecule, in which the π-electrons are delocalized for the entire length of the polyene chain. This feature is responsible for the molecular shape, chemical reactivity, light-absorbing properties, and hence the color of carotenoids [1]. Two geometric configurations, *cis* or *trans* isomers, can exists for each double bond in the polyene chain. Most carotenoids are present in nature in the *trans* form [2]. About 750 different carotenoids (without counting their geometrical isomers) have been isolated and identified from natural sources, and some of them are very important to human health thanks to their antioxidant properties. Carotenoids are counted among the major groups of phytochemicals that may contribute to the total antioxidant capacity (TAC) of plant foods [3]. Among the 600 carotenoids in nature, only 40 are present in the human diet, and about 20 of these have been identified in human blood and tissue. The most common are α-carotene, β-carotene, lycopene, lutein, zeaxanthin, and cryptoxanthin.

There are several reaction mechanisms that allow carotenoids to exercise their antioxidant functions. The first mechanism that was clearly described is the ability of carotenoids to quench a highly reactive form of oxygen known as singlet oxygen. Many studies report on the ability of various carotenoids to interfere with radical-initiated reactions, particularly with those that result in lipid peroxidation [4]. Carotenoids react with free radicals thanks to the electron transfer mechanism or possible addition reactions. In this way, carotenoids protect cells from damage and oxidative stress, and are also able to boost immune function [5,6].

Epidemiological studies suggest that a diet high in fruit and vegetables is associated with decreased incidence of cancer, cardiovascular disease, and probably of other degenerative or age-related diseases. In fact, fiber, vitamins, phytosterols, sulfur compounds, organic acids, polyphenols, and carotenoids are contained in plant foods and contribute to their health effects [7]. As mentioned above, carotenoids are excellent antioxidants, and a limited number of them are the molecular precursor of vitamin A. Vitamin A is an essential nutrient for humans; pro-vitamin A carotenoids are partly converted to retinol by the oxygenase and reductase enzymes, and the retinol so produced is available for absorption [8].

However, the potential beneficial effects of these bioactive molecules are strictly limited by their bioavailability. There are many factors that influence phytochemical bioavailability, such as the class of the compounds, molecular mass, polarity, solubility, stability, isomerization, associations with the plant matrix, and digestion by gastrointestinal enzymes [9]. The Food and Drug Administration (FDA) has defined bioavailability as the rate and extent to which the active substances or therapeutic moieties contained in a drug are absorbed and become available at the site of action [10]. This definition is also extended to active compounds present in foods. A prerequisite for absorption is the release from the food matrix and solubilization during digestion, which is commonly indicated by the term bioaccessibility [11]. Bioaccessibility includes all events that occur during food digestion, but excludes absorption and assimilation through epithelial tissue and presystematic metabolism. This last concept is incorporated in another definition that is bioactivity. It evaluates how bioactive compounds are transported and arrive at the target tissue, how they interact with other molecules, the biotrasformations that can occur, the generation of biomarkers, and the physiological response induced. Bioaccessibility and bioactivity are part of a unique term, bioavailability [12]. It is clear that a multidisciplinary approach from medicine, food and analytical chemistry, and nutraceutical studies is necessary to obtain a complete description and relevant results.

There are several studies that analyze the composition of carotenoids in different species and varieties of pumpkins, emphasizing that there are high concentrations of these compounds closely related to the varieties or growing conditions [13,14,15]. It is common that many vegetables are cooked by a simple boiling, steaming, baking, or microwave process before being consumed. These cooking methods would involve a series of changes in the physical characteristics and chemical composition of vegetables, which can take place consecutively or simultaneously. Many studies focused attention on the isomerization and/or degradation of these compounds, but it is known that both positive and negative effects are possible with regard to cooking conditions, chemical structure, and characteristics of the vegetable matrix [16]. For this reason, the investigation of the concentrations of a target compound before and after a cooking process is essential for bioaccessibility studies. Carotenoid absorption and metabolism have been comprehensively investigated [9,17,18]. Generally, the absorption of lipophilic compounds includes several passages: the mechanical and enzymatic disruption of food matrix, release and emulsification into lipid droplets in the stomach followed by incorporation into mixed micelles, and transition through intestinal cells where they are packed into chylomicrons and secreted into the lymphatic system.

Considering what has been mentioned above, the main objectives of this study are: (1) characterization of major carotenoids in two varieties of pumpkins, commonly cultivated in the northeast of Italy; (2) evaluation of the effects of two different cooking methods on the concentration of lutein and β-carotene; (3) investigation of the bioaccessibility of the two selected compounds.

## 2. Results and Discussion

### 2.1. Carotenoid Determination by HPLC-UV/Vis-APCI-MS/MS

There are many factors that can influence biosynthesis and metabolism and modify the concentration of bioactive compounds in fruits and vegetables [19]. Major qualitative and quantitative differences in carotenoids can be noted in relation to cultivar, environmental conditions (temperature, nutrient availability, intensity of sunlight, ripening stage, harvest time), and genetic factors. The composition of carotenoids in the raw pumpkins was determined by reverse-phase HPLC. In this study, a C30 column was used because the recent literature showed that this type of column is the most suitable for carotenoid separations [20,21]. This stationary phase exhibits enhanced selectivity towards *cis–trans* carotenoids compared to C18 ligands [22]. Usually in reverse-phase analysis, the sequence of chromatographic peaks reflects a decreasing polarity of eluted compounds, and more polar xanthophylls are eluted first. However, on a C30 column the length of the polyene chain is the essential parameter for the observed retention time and isomer separation [20]. Even if there are small differences in the molecular structure, significant differences in retention times were observed.

**Figure 1:**
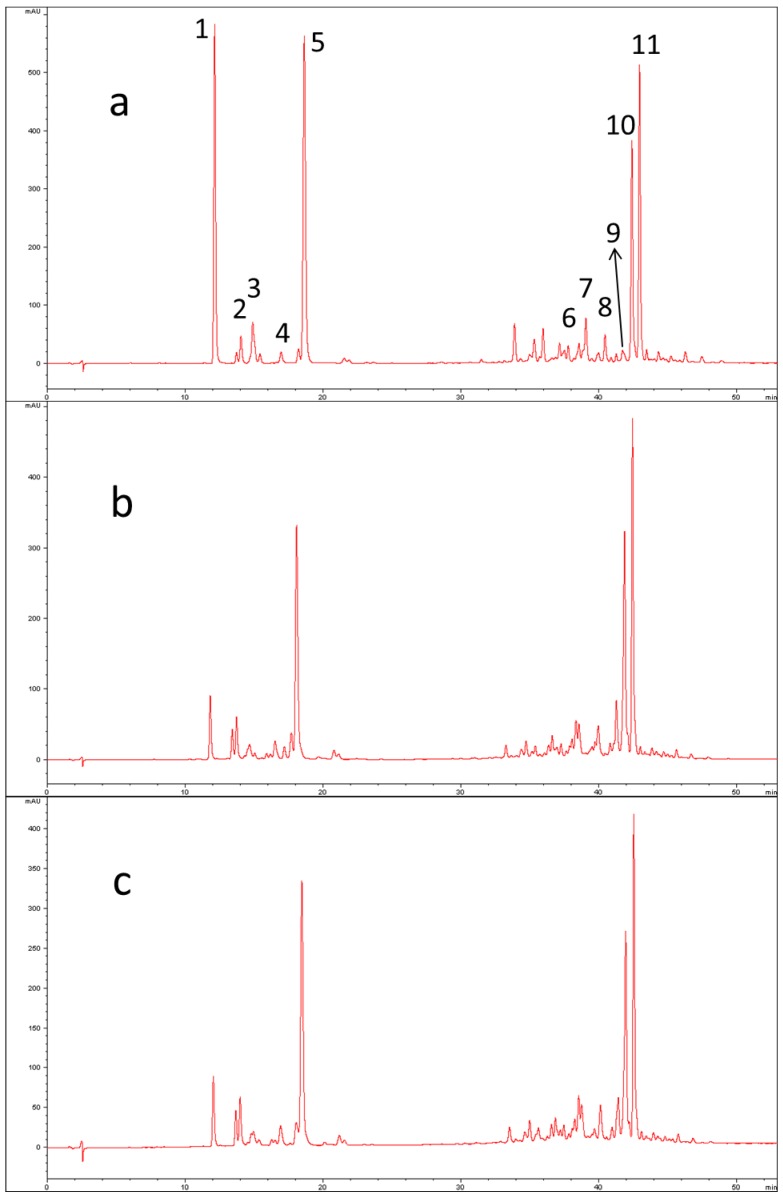
HPLC-UV/Vis chromatograms of “Violina” pumpkin extracts: (**a**) raw fresh sample; (**b**) steam-cooked sample; (**c**) oven-cooked sample.

**Figure 2:**
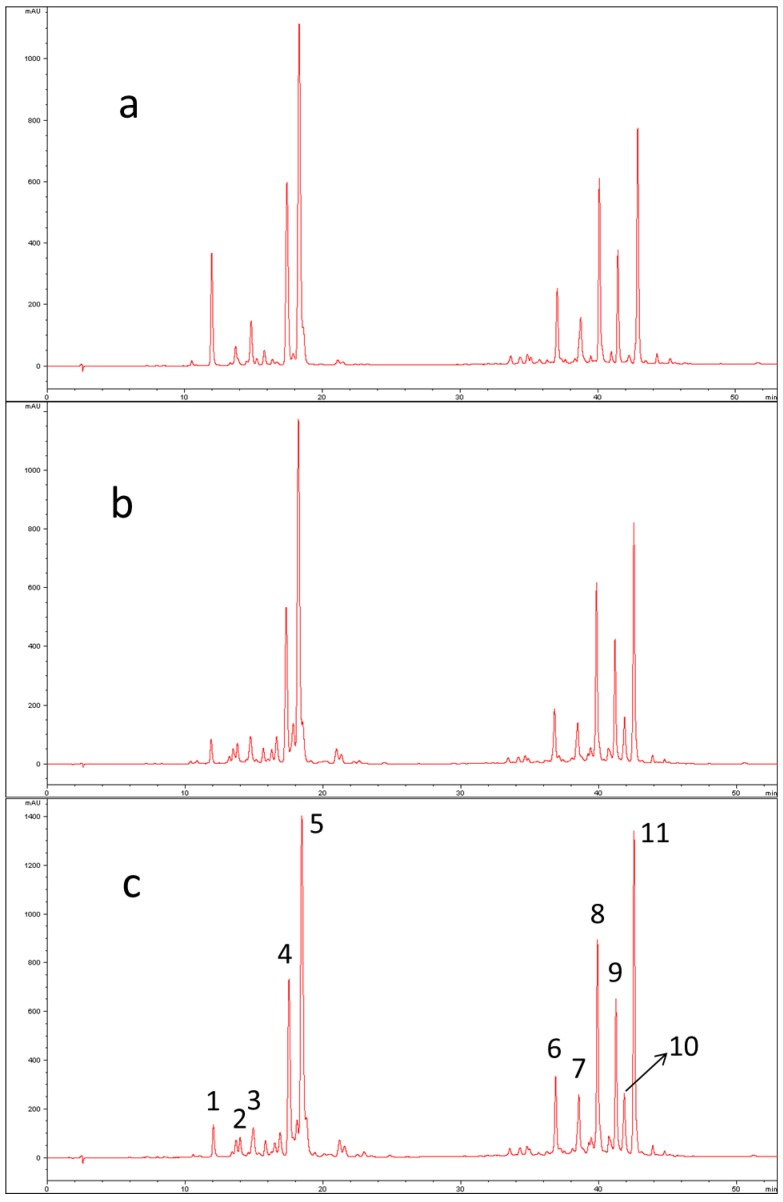
HPLC-UV/Vis chromatograms of “Delica” pumpkin extracts: (**a**) raw fresh sample; (**b**) steam cooked sample; (**c**) oven cooked sample.

The UV-Vis parameters used for the identification of peaks are shown in Table 1 together with their chromatographic retention and positive APCI-MS/MS data (protonated molecular ion and its fragment ions). Usually, UV/Vis carotenoid spectra show three relative maxima. Wavelengths are reported in the fourth column of Table 1. The %III/II data represent ratios between the third and second relative maxima for each chromatographic peak. Peak numbers refer to those chromatographic bands identified in Figure 1a and Figure 2c. It was possible to tentatively identify seven peaks out of eleven by means of both UV-Vis and MS data, whereas for Peak 3 the proposed identification was made possible only on the basis of spectrophotometric and literature data because no clear MS and MS/MS spectra were obtained.

The first group of chromatographic peaks includes epoxy- and hydroxy-carotenoids, followed by the second group that consists of nonpolar carotenes. Peaks 5 and 11 were assigned to lutein and β-carotene, respectively, using analytical standards. For other peaks, a tentative identification was made based on comparison of the parameters with the literature data.

Peak 4 has very low intensity in the chromatogram of *C. moschata* “Violina”, while it is more abundant in *C. maxima* “Delica”. The opposite scenario occurs for Peak 1, even if its intensity is higher in raw pumpkin than cooked. By comparing with other published works concerning analysis of other pumpkin varieties, it can be hypothesized that the signal for Peak 1 is attributable to violaxanthin, while Peak 4 refers to zeaxanthin. Azevedo-Meleiro and Rodriguez-Amaya (2007) do not identify violaxanthin in the *C. moschata* “Menina Brasileira”, while Provesi et al. (2011) identify the signal both in the *C. moschata* “Menina Brasileira” and in the *C. maxima* “Exposicao” but the quantification is smaller in the first one. On the contrary, peak 10 is clearly present in the chromatographic profile of *C. moschata*, while it can not be evidenced in raw *C. maxima*. Literature data show that α-carotene is absent or present in extremely low concentration in the *C. maxima* [14,15]. For this signal, $\lambda_{max}$ and %III/II registered are very similar to tabulated references and it can be hypothesized that it corresponds to α-carotene [14,23]. The parameter % III/II is the ratio of the height of the longest wavelength absorption peak, designated III, and that of the middle absorption peak, designated as II, taking the minimum between the two peaks as baseline [24].

### 2.2. Carotenoid Levels in Raw and Cooked Pumpkins

Concentrations of carotenoids in raw pumpkins are different from those reported in other studies regarding the same varieties and similar cultivars of pumpkins [25]. An external calibration method was applied for quantitative purposes. Area vs. concentration data have been fitted with linear regression for the two standards, obtaining correlation coefficients higher than 0.995. Table 2 reports the amount of lutein and β-carotene in samples. Quantitative data have been obtained by means of UV/Vis detector with external calibration method, whereas APCI-MS/MS was hyphenated for qualitative purposes aimed at molecular identification of carotenoids.

Azevedo-Meleiro and Rodriguez-Amaya (2007) detected concentrations of 66.7 ± 9.1 μg/g f.m. (fresh matter) all-*trans*-β-carotene in the *C. moschata* “Menina Brasileira’, and of 15.4 ± 4.2 μg/g f.m. for *C. maxima* “Exposicao”. Lutein was also slightly higher in the former than in the latter, with values of 17.4 ± 3.5 vs. 10.7 ± 3.9 μg/g f.m., respectively [15]. On the contrary, Provesi et al. (2011) noted that lutein levels were higher in *C. maxima* “Exposicao” than *C. moschata* “Menina Brasileira” (10.43 ± 0.13 vs. 0.59 ± 0.18 μg/g f.m.), while the concentration of all-*trans*-β-carotene was similar between the two varieties, but lower than previous cited work [14]. Different factors may be responsible for the different detected concentrations. It is well known that climate has a significant influence on the content of carotenoids in vegetables. Fruits of the same cultivars produced in different regions exhibit higher or lower carotenoid concentrations in relation to warmer or a more temperate climates. It is probably connected with the increase of carotenogenesis, when fruits are more exposed to sunlight, even if it may cause photodegradation. Table 2 not only reports the amount of lutein and β-carotene in the two fresh raw cultivars, but it also compares absolute determination calculated as lutein-equivalents and β-carotene-equivalents with relative quantification (area %). This means that, in the first case, all other carotenoids were quantified by using a calibration curve obtained for lutein (Peaks 1 to 4) and for β-carotene (Peaks 6 to 10). In the second case, on the other hand, each peak area was reported as a percentage of the total area.

Pumpkin pulp is commonly consumed cooked and, in Italy, baked, and steamed pumpkins are particularly widespread. In this study, the two cooking methods are compared in terms of carotenoid content. The only difference between the two methods was temperature: 200 ${}^{\circ }$C for oven-cooked samples, and 100 ${}^{\circ }$C for steamed, while time was kept constant (20 min). However, the two cooking methods caused different loss of humidity; to make a direct comparison between raw and cooked samples, the percentage of dry matter was calculated. Data as μg/g of dry matter are reported in Table 3. The percentage loss of content both in lutein and in β-carotene was less than 10% in the steam-cooked pumpkins, while it was about 30% in the oven-cooked pumpkins. Therefore, steam cooking was outlined as the optimal method, and this is probably due to lower thermal shock suffered compared to oven cooking. This is partially in agreement with previous studies that explain that heat treatment improves the bioavailability of carotenoids. Cooking practices break down food matrices and loosen carotene-binding fibers, but the bioavailability and, sometimes, the carotene content could increase [26].

### 2.3. Carotenoid Bioaccessibility

It is well known that the absorption of hydrophobic molecules depends upon efficient release from the food matrix and subsequent solubilization by bile acids and digestive enzymes, culminating in their incorporation into mixed micelles [27]. Micellarization is required for the cellular uptake of carotenoids. In vitro digestion models were used to investigate the structural changes, modifications, digestibility, and release of components during simulated gastrointestinal digestion. Depending on the type of study performed, it is evaluated whether to follow a static or dynamic in vitro model. In vitro dynamic digestion is closest to in vivo conditions: it can simulate gastric emptying rate, peristaltic movements, gradual pH changes in different compartments, and transit time through the small intestine [9]. Unfortunately, this method requires complex computer-controlled systems, higher operating costs, and more time. On the other hand, static digestion takes shape as a more flexible and less expensive model. It is particularly indicated for a large number of samples or to assess many experimental conditions. Unfortunately, static models do not evaluate the dynamic physiological response to the introduction of a food bolus. In both cases, for the analysis of lipophilic compounds, a separation of the micellar fraction is required before investigation: ultracentrifugation for the static model; filtration or employing a membrane for a dynamic model.

**Figure 3:**
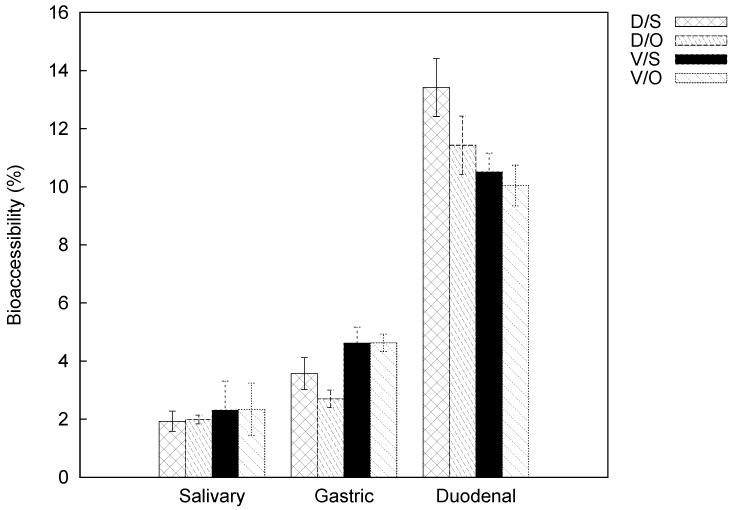
Bioaccessibility of lutein from cooked pumpkins: D/S, “Delica” steam-cooked; D/O, “Delica” oven-cooked; V/S, “Violina” steam-cooked; V/O, “Violina” oven-cooked.

**Figure 4:**
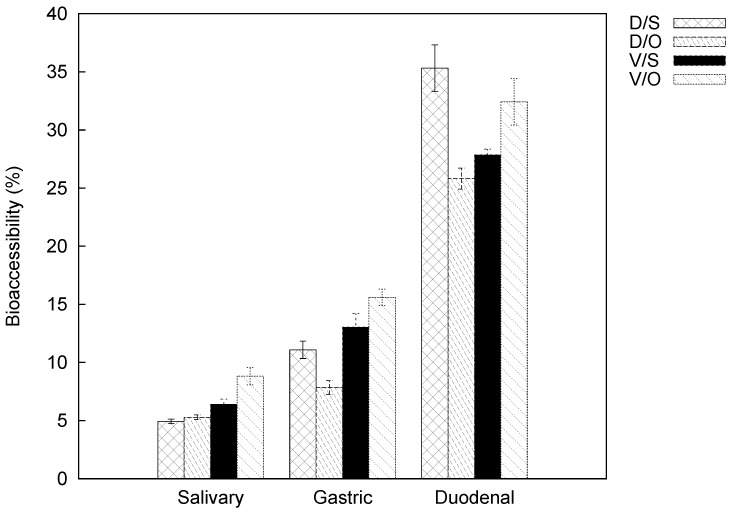
Bioaccessibility of β-carotene from cooked pumpkins: D/S, “Delica” steam-cooked; D/O, “Delica” oven-cooked; V/S, “Violina” steam-cooked; V/O, “Violina” oven-cooked.

A quantitative determination of carotenoids is required for a correct estimation of bioaccessibility. For this purpose, bioaccessibility is calculated as the percentage of the present quantities in the exhaustive extracts of fresh matter, for each variety and each cooking method (see Table 2 and Table 3). The bioaccessibility results for lutein and β-carotene from oven- and steam-cooked samples and for each cultivar are reported in Figure 3 and Figure 4, respectively. Samples were subject to simulated digestion through the salivary, gastric, and duodenal steps.

The bioaccessibility values of lutein and β-carotene progressively increase from the salivary to the duodenal phase for both pumpkin varieties and cooking methods. Our findings revealed that the bioaccessibility of lutein is always lower than that of β-carotene. This evidence is not attributable to the lower lutein content in the initial matrix, but rather to possible food-related factors that influence bioaccessibility itself. Bioaccessibility values for lutein in the salivary phase are similar and the average is 2.14% (data changed from 1.93% to 2.34%), while for β-carotene they were more heterogeneous (data changed from 4.94% to 8.83%). Both compounds were less bioaccessible for ‘Delica’ than ‘Violina’ regardless of cooking mode. Comparable behavior can be evidenced in the gastric phase: lutein values were similar and lower than 5% (from 2.70% to 4.63%), while β-carotene did not have a well-defined average value (from 7.83% to 15.60%). In this case, bioaccessibility was also higher for Violina both in oven and steam-cooked samples. In the duodenal phase, lutein values ranged from 10.04% to 13.42%, whereas for β-carotene from 25.81% to 35.32%.

The calculation of the difference in bioaccessibility data between the gastric and salivary phases shows values of about 2 for lutein and 6 for β-carotene (for both compounds, the gap was smaller in the case of the oven-cooked Delica). The same calculation between the other two phases underlined that bioaccessibility in the duodenal phase is more than twice the gastric values (data are lower than 10 for lutein and 25 for β-carotene).

A global examination of the bioaccessibility results shows that the release of lutein and β-carotene was higher during the duodenal phase and also that cooking method did not affect the release of carotenoids from the initial matrix. All of these aspects require further investigation, paying attention to all the causes that influence bioaccessibility of carotenoids.

## 3. Materials and Methods

### 3.1. Chemicals

Ultrapure water was obtained by a Milli-Q purification system (Millipore, Bedford, MA, USA). Hexane, methanol, and acetone from Sigma-Aldrich (St. Louis, MO, USA) were used for the extraction. Methanol (HPLC grade), acetonitrile (HPLC grade), methyl tert-butyl ether (HPLC grade), purchased from Sigma-Aldrich (St. Louis, MO, USA), were employed for high-performance liquid chromatographic analyses. Pepsin from porcine gastric mucosa (≥400 U/mg), α-amylase from *Bacillus licheniformis* (≥500 U/mg), bile salts (microbiology grade), and pancreatin from porcine pancreas (USP grade) were purchased from Sigma Aldrich Co. (St. Louis, MO, USA). The lutein and β-carotene standards were obtained from Extrasynthese (Genay, France).

### 3.2. Instrumentation

An ALC multispeed refrigerated centrifuge (PK121R) was purchased from Thermo-Scientific (Waltham, MA, USA). An Ultraturrax (T18 basic) was obtained from IKA (Staufen im Breisgau, Germany). HPLC-UV/Vis-APCI-MS/MS analyses were performed with a modular Agilent liquid chromatographer (1100 series) equipped with a binary pump, a degasser, an autosampler injector, a column oven, and a photodiode array detector. The outstream of the detector high-pressure flow-cell was online coupled with a Thermo Scientific LTQ XL linear ion trap mass spectrometer through an APCI ion source.

### 3.3. Reagent Preparation

Electrolyte stock solutions were prepared at the following concentrations: KCl 0.5 M; KH${}_{2}$PO${}_{4}$ 0.5 M; NaHCO${}_{3}$ 1 M; NaCl 2 M; MgCl${}_{2}$·(H${}_{2}$O)${}_{6}$ 0.15 M; (NH${}_{4}$)${}_{2}$CO${}_{3}$ 0.5 M; CaCl${}_{2}$·(H${}_{2}$O)${}_{2}$ 0.3 M.

These solutions were used to simulate biofluids involved in the gastrointestinal digestion process and build an in vitro model. Three different solutions were prepared as reported by Minekus [27,28]:

Simulated salivary fluid (SSF): 15.1 mL of KCl; 3.7 mL of KH${}_{2}$PO${}_{4}$; 6.8 mL of NaHCO${}_{3}$; 0.5 mL of MgCl${}_{2}$; 0.06 mL of (NH${}_{4}$)${}_{2}$CO${}_{3}$. Simulated gastric fluid (SGF): 6.9 mL of KCl; 0.9 mL of KH${}_{2}$PO${}_{4}$; 12.5 mL of NaHCO${}_{3}$; 0.4 mL of MgCl${}_{2}$; 0.5 mL of (NH${}_{4}$)${}_{2}$CO${}_{3}$; 11.8 mL of NaCl. SGF was adjusted to pH = 3 with HCl 1 M. Simulated intestinal fluid (SIF): 6.8 mL of KCl; 0.8 mL of KH${}_{2}$PO${}_{4}$; 85 mL of NaHCO${}_{3}$; 0.33 mL of MgCl${}_{2}$; 38.4 mL of NaCl. SIF was adjusted to pH = 7 with HCl 1 M.

Enzyme solutions were freshly prepared and preincubated at 37 ${}^{\circ }$C before use. α-amylase 1500 u/mL: 30 mg of enzyme in 20 mL of SSF. Pepsin 20,000 u/mL: 600 mg of enzyme in 20 mL of SGF. Pancreatin 800 u/mL: 320 mg in 40 mL of SIF. Bile salts: 625 mg in 25 mL of SIF. Purchased enzymes were evaluated according to reference tests as specified in the literature and by the manufacturers [27]: (a) the determination of α-amylase activity was done with an enzymatic assay, which was based on spectrophotometric stop reaction using soluble potato starch as a substrate; (b) pepsin activity assay was based on spectrophotometric stop reaction using hemoglobin as the substrate; (c) pancreatin activity was evaluated for its trypsin and chimotrypsin activity based on continuous spectrophotometric rate determination using *p*-toluene-sulfonil-l-arginine methyl ester (TAME) and *N*-benzoyl-l-tyrosyne ethyl ester (BTEE) as substrates.

### 3.4. Sample Collection and Preparation

The two pumpkin cultivars under study were produced in a well-defined area in the southern part of Po Delta (Massenzatica, Ferrara, Italy). Samples were collected from randomized fields in November 2016, and 10 mature pumpkins of each variety were sampled for the analysis. *Cucurbita maxima* ’Delica’ weighs approximately 2 kg and has a 12–16 cm longitudinal and a 20–25 cm transverse diameter. It is spherical but flattened at both extremities, and it has a dark-green peel and a light-orange pulp. *Cucurbita moschata* ‘Violina’ is 45–55 cm long, with an 8–12 cm transverse diameter in the cylindrical portion and a 15–20 cm transverse diameter in the bulbous portion, and weighs approximately 3 kg. For sample preparation, *C. maxima* ‘Delica’ was divided into four parts by two longitudinal cuts, while *C. moschata* ‘Violina’ was quartered longitudinally and transversely, resulting in two bulbous and two cylindrical portions. For each pumpkin, peel and seeds were removed and the pulp was cut into small pieces, mixed and stored at −20 ${}^{\circ }$C until analyzed. For analyses, samples were collected from each section and homogenized in a food multiprocessor to obtain a homogeneous mass.

### 3.5. Cooking Methods

In this work, two different cooking methods were considered. Refrigerated and raw pieces were collected from every section of pumpkins, and were then oven-cooked (200 ${}^{\circ }$C for 20 min) or steam-cooked (100 ${}^{\circ }$C for 20 min). For analyses, cooked samples were placed in a food multiprocessor to obtain a homogeneous samples.

### 3.6. Carotenoids Extraction

Carotenoids were extracted from the (raw and cooked) samples using a modified literature procedure by H. S. Lee et al. [22] and C. Kurz et al. [29]. The extraction procedure was checked with regard to structural modifications or damage. The extraction procedure had to be adapted to the matrix being analyzed because carotenoids are found in a considerable variety of foods. The chosen solvent should efficiently extract all carotenoids present in the sample. For this reason pumpkin samples were finely grinded before extraction.

About 5 g of pumpkin puree (raw and cooked), obtained as previously described, was extracted with 15 mL of a mixture of hexane/methanol/acetone (2:1:1, *v*/*v*/*v*) in a centrifuge tube (50 mL). The extraction was performed at room temperature for 30 min under continuous and slight shaking. The sample was centrifuged (9000 rpm at 5 ${}^{\circ }$C) for 5 min and the supernatant was recovered. This step was repeated in sequence twice more, and the collected organic portions were evaporated at a temperature lower than 30 ${}^{\circ }$C using a rotary evaporator. The dried extracts were dissolved in 4 mL of acetonitrile and filtered (nylon, 0.22 μm) before HPLC analyses. According to the literature data [14], saponification was not performed because it causes loss in carotenoid content, especially xanthophylls, which are in lower concentrations in this matrix. All procedures were carried out with as dim a light as possible.

### 3.7. In Vitro Static Digestion Model

The in vitro digestion model and the composition of simulated fluids were adapted from earlier studies as described in Section 3.3 and below [27,30]. The mouth, stomach, and small-intestine (duodenum) phases were evaluated and simulated using the three fluids reported above(SSF, SGF, and SIF). Digestion simulation was performed with a thermostatic shaker at a physiological temperature of 37 ${}^{\circ }$C. Water and all reagent solutions were warmed at 37 ${}^{\circ }$C before and during use. Analyses were carried out on the cooked samples of both varieties of pumpkins and for both cooking methods.

*Salivary phase*—About 3 g of the homogenized matrix were mixed with 3.5 mL of SSF, 975 μL of water, 25 μL of CaCl${}_{2}$ and 500 μL of α-amylase solution. Sample was vortex-mixed for 1 min, centrifuged (9000 rpm at 5 ${}^{\circ }$C) for 5 min, and then an aliquot of 2.5 mL was obtained for the analysis.

*Gastric phase*—Sample from the salivary phase was added with 7.5 mL of SGF, 295 μL of water, 5 μL of CaCl${}_{2}$, 200 μL of HCl 1 M, and 2 mL of pepsin solution. After vortex-mixing for 30 s, pH was measured and readjusted with HCl 1 M if it was higher than 3. Samples were incubated for 2 h at 37 ${}^{\circ }$C in the thermostatic shaker. Then, samples were centrifuged (9000 rpm at 5 ${}^{\circ }$C) for 5 min and 5 mL of each solutions was collected.

*Intestinal phase*—The sample from the previous phase was mixed with 11 mL of SIF, 3.61 mL of water, 40 μL of CaCl${}_{2}$, 150 μL of NaOH 1 M, 5 mL of pancreatin solution, and 0.2 mL of bile salts solution. It was vortexed for 30 s, and the pH was measured and readjusted with NaOH 1 M if it was not close to the value of 7. Samples were then incubated for 2 h at 37 ${}^{\circ }$C. At the end of the intestinal phase, samples were centrifuged (9000 rpm at 5 ${}^{\circ }$C) for 5 min and 5 mL of solutions was collected.

#### Carotenoid Extraction from Digesta

At the end of each simulated gastrointestinal step, the micellar fraction was separated using the method proposed by E. Biehler et al. with some modifications [31]. Aliquots from different digesta were separately conveyed to centrifuge tubes, and the following extraction procedure was repeated twice. Carotenoids were extracted by the addition of 2.5 mL (in the case of the salivary phase) or 5 mL (in the case of other two phases) of a mixture of hexane/methanol/acetone (2:1:1, *v*/*v*/*v*). Samples were vortexed for 1 min, centrifuged (9000 rpm at 5 ${}^{\circ }$C) for 5 min, and finally the supernatant was recovered. The combined hexane phases were dried as reported in Section 3.6. The dried extracts were dissolved in 1 mL of acetonitrile and filtered (nylon, 0.22 μm) before HPLC analyses.

### 3.8. HPLC-UV/Vis-APCI-MS/MS Analysis

Chromatographic separation was performed on a C30 Develosil RP-Aqueous by Phenomenex (Torrance, CA, USA) 150 × 3.0 mm column, packed with 3.0 μm particles and a porosity of 140 Å. Mobile phases were mixtures of water and acetonitrile ( 70:30 *v*/*v*; channel A), and methanol: methyl tert-butyl ether ( 50:50 *v*/*v*; channel B). Eluent composition changed from 20% to 47% of channel B in 10 min, from 47% to 57% of channel B in 10 min, from 57% to 100% of channel B in 20 min, followed by 100% channel B isocratic for 13 min: total run time was 53 min. After each analysis, the column was re-equilibrated at the starting conditions. The running flow rate was 0.4 mL/min, and the column temperature was set at 23 ${}^{\circ }$C to optimize carotenoid isomer separation by the column used [32]. The quantity of injected sample was 5 μL and all carotenoids were monitored at 450 nm. Additionally, UV-Vis spectra were obtained between 200 and 600 nm at a spectral acquisition rate of 0.5 scans/s (peak width 0.03 min). HPLC-UV/Vis was coupled in the series with an APCI-MS/MS mass spectrometer. APCI was operated in positive ionization mode and under normal scan condition (10 msec scan rate for MS and 100 msec scan rate for MS/MS). The following source parameters were also used: corona discharge 4–6 μA; vaporizer temperature 400 ${}^{\circ }$C; capillary temperature 300 ${}^{\circ }$C; capillary voltage 45 V; tube lens voltage 85 V.

## 4. Conclusions

In this work, two main pumpkin varieties from the northeast of Italy were investigated in terms of their carotenoid resistance to two different cooking methods. Pumpkins are employed as cooked food to prepare processed typical foodstuffs (mainly pasta). Nowadays, some of these agrofood products have a protected geographical indication (PGI) from the European Community, whereas the same does not happen for fresh raw material such as pumpkins. This is the reason why the authors consider it is of fundamental importance to also trace the production origin of fresh ingredients. Thus, this study was aimed at preliminarily evaluating if selected carotenoids could be considered as traceability markers that are able to aid in the identification of different cultivars even after cooking. This can be considered a first step towards using molecular markers in cooked vegetables employed in processed food. Additionally, the bioaccessibility of two selected carotenoids was studied by means of a static in vitro gastrointestinal digestion process. Obtained results evidenced that, within the general trend where bioaccessibility progressively increases from the salivary step to the duodenal, cooking method can influence bioaccessibility data, particularly during the duodenal stage: the “Delica” pumpkin showed the latest bioaccessibility for both lutein and β-carotene when it has been steam–cooked; on the contrary, “Violina” provided for comparable bioaccessibility for lutein regardless of cooking method, while β-carotene displayed larger bioaccessibility when oven–cooked.

## Figures and Tables

**Table 1:** Chromatographic, spectroscopic, and APCI–MS/MS mass spectrometric parameters of the tentatively identified carotenoid peaks (see Figure 1 and Figure 2).

Peak	Retention Time (min)	Compound	$\lambda_{\max}$	% III/II	[M + H]${}^{+}$ (*m*/z)	Fragment Ions (*m*/z)
1	12.08	violaxanthin	418,442,470	87	601	534, 583, 429
2	13.83	astaxanthin	418,442,470	82	597	579, 285, 379
3	15.00	antheraxanthin	426,448,474	27		
4	17.61	zeaxanthin	422,447,476	53	569	551, 489, 477, 416
5	18.54	lutein	422,446,474	53	569	495, 459, 430, 477
6	37.15	lycopene	419,442,470	72	537	457, 413, 177
7	38.85		420,442,470	58		
8	40.20		422,446,474	54		
9	41.54		422,446,474	53		
10	42.18	α-carotene	422,446,474	51	537	457, 413, 177
11	42.85	β-carotene	425,452,477	18	537	457, 445, 413, 400, 269, 177

**Table 2:** Quantification of known and unknown chromatographic peak in fresh raw pumpkins. For each peak determination was reported: as absolute quantification (μg/g of fresh matter), by using external calibration curve of lutein (peaks from 1 to 5) and of β-carotene (peaks 6 to 11); as relative quantification in terms of peak area%.

Peak	Absolute Quantification		Relative Quantification	
	*C. maxima* (Delica)	*C. moschata* (Violina)	*C. maxima* (Delica)	*C. moschata* (Violina)
violaxanthin	9.09±0.99	10.53±0.96	7.24±0.42	24.72±1.04
astaxanthin	2.54±0.39	1.18±0.06	1.70±0.04	1.73±0.10
antheraxanthin	4.34±0.74	1.73±0.14	3.21±0.07	3.08±0.09
zeaxanthin	16.99±3.08	nd	13.83±0.21	nd
*lutein*	37.12±3.08	10.30±1.01	30.74±0.10	24.12±0.65
lycopene	19.25±2.69	6.44±0.24	5.08±0.03	2.38±0.09
7	17.79±2.42	7.55±0.30	4.58±0.03	3.49±0.18
8	38.73±6.09	6.89±0.40	11.71±0.06	2.82±0.02
9	23.66±3.28	6.39±0.61	6.59±0.02	2.31±0.29
α-carotene	nd	17.56±2.26	nd	13.40±0.41
*β-carotene*	49.29±3.78	26.22±2.23	15.32±0.08	21.95±1.44

**Table 3:** Quantification of lutein and β-carotene in cooked pumpkin samples. Data are reported as μg/g.

	*C. maxima* (Delica)		*C. moschata* (Violina)	
	Lutein	β-carotene	Lutein	β-carotene
Oven-cooked	41.18±1.39	58.46±1.85	11.22±0.08	25.71±2.36
Steam-cooked	39.09±0.35	47.00±3.06	10.86±0.23	31.99±0.48
